# Supplementary material for: Emerging spin–phonon coupling through cross-talk of two magnetic sublattices
Source: Nat Commun. 2022 Jan 21;13:443. doi: 10.1038/s41467-021-27267-8 (PMC8783005; doi:10.1038/s41467-021-27267-8)
Supplement: Supplementary file 1 — Supplementary Information [file 41467_2021_27267_MOESM1_ESM.pdf]

Supplementary Information:

Emerging spin-phonon coupling through cross-talk of two magnetic sublattices

Mads C. Weber[1,2,3,4]*, Mael Guennou[2,3], Donald M. Evans[5], Constance Toulouse[2,3], Arkadiy Simonov[1], Yevheniia Kholina[1], Xiaoxuan Ma[6], Wei Ren[6], Shixun Cao[6,§], Michael A. Carpenter[5], Brahim Dkhil[7], Manfred Fiebig[1] and Jens Kreisel[2,3]

[1]*Department of Materials, ETH Zurich, 8093 Zurich, Switzerland.*

[2]*Department of Physics and Materials Science, University of Luxembourg, 41 Rue du Brill, L-4422 Belvaux, Luxembourg*

[3]*Materials Research and Technology Department, Luxembourg Institute of Science and Technology, 41 Rue du Brill, L-4422 Belvaux, Luxembourg*

[4]*Institut des Molécules et Matériaux du Mans, UMR 6283 CNRS, Le Mans Université, Le Mans 72085, France*

[5]*Department of Earth Sciences, University of Cambridge, Downing Street, Cambridge CB2 3EQ, United Kingdom*

[6]*Department of Physics, Materials Genome Institute and International Center for Quantum and Molecular Structures, Shanghai University, Shanghai, 200444, China*

[7]*Laboratoire Structures, Propriétés et Modélisation des Solides, Centrale Supélec, CNRS-UMR8580, Université Paris-Saclay, 91190 Gif-sur-Yvette, France*

**mads.weber@univ-lemans.fr*

[§]*sxcao@shu.edu.cn*

Content

Supplementary Note 1: Evolution of the Raman spectra with temperature 2

Supplementary Note 2: Evolution of the frequencies and the FWHM of phonon modes with temperature 3

Supplementary Note 3: New phonon bands in the low temperature regime 5

Supplementary Note 4: RUS analysis of the relaxation processes within the spin reorientation 6

Supplementary Note 5: Primary RUS spectra tracing the spin reorientation transition 8

Supplementary Note 6: Relaxation during the spin reorientation of further elastic resonances........ 9

Supplementary Note 7: Magnon evolution with temperature 10

Supplementary Note 8: RUS analysis for the low temperature evolution........ 11

Supplementary Note 9: Temperature evolution of the lattice parameters........ 13

Supplementary References 15

Supplementary Note 1: Evolution of the Raman spectra with temperature

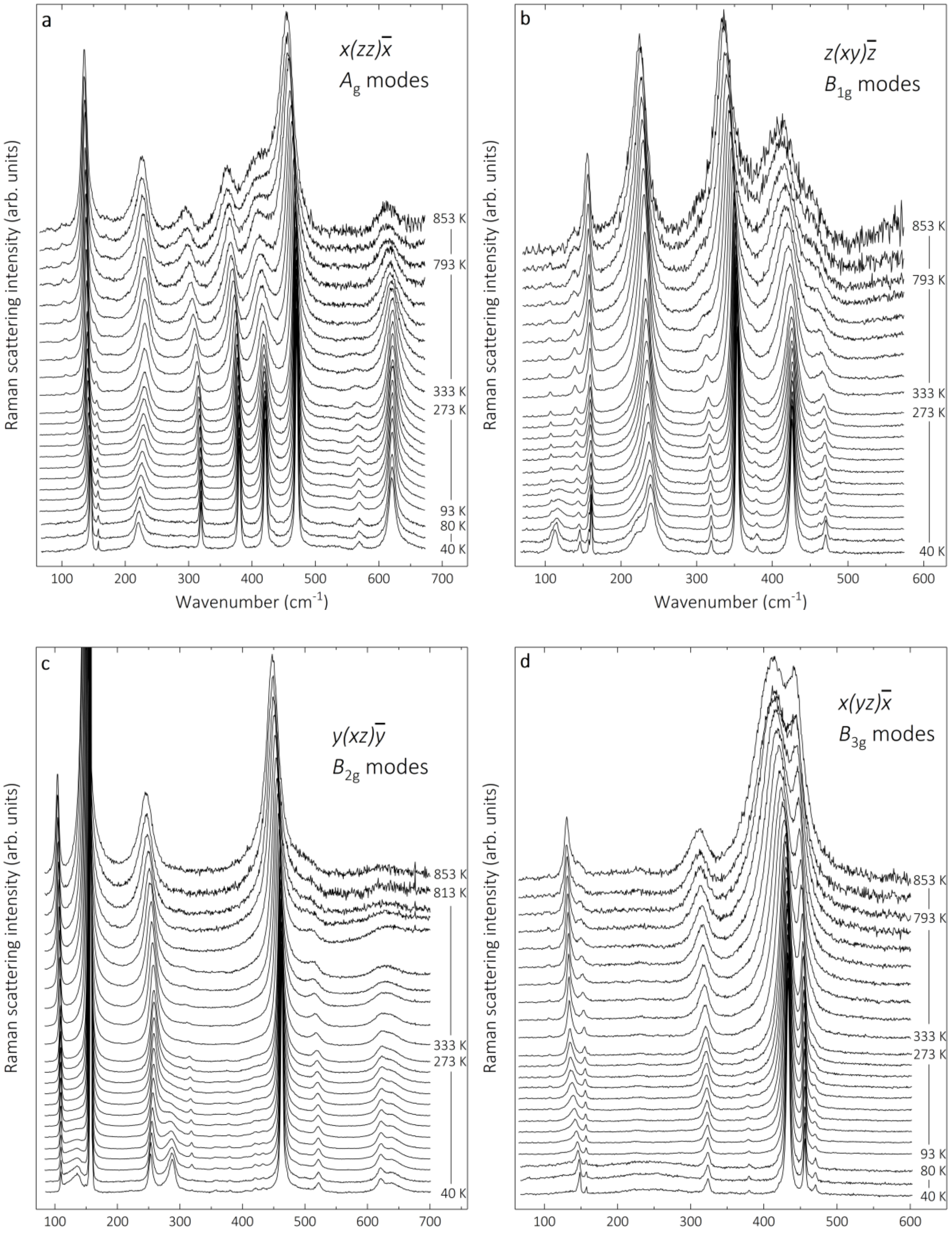

Supplementary Fig. 1 Evolution of the polarized Raman spectra of $SmFeO_3$ with temperature. A_g, B_{1g}, B_{2g}, B_{3g} modes are given in **a**, **b**, **c**, **d**. The Raman spectra were corrected for thermal occupation by (n + 1), where n is the Bose-Einstein occupation number. For better visibility, we normalized the spectra to the peak intensities of the modes at 470 cm^{-1}, 353 cm^{-1}, 462 cm^{-1} and 433 cm^{-1} for the spectra of A_g, B_{1g}, B_{2g} and B_{3g} modes, respectively (values at 40 K).

Supplementary Note 2: Evolution of the frequencies and the FWHM of phonon modes with temperature

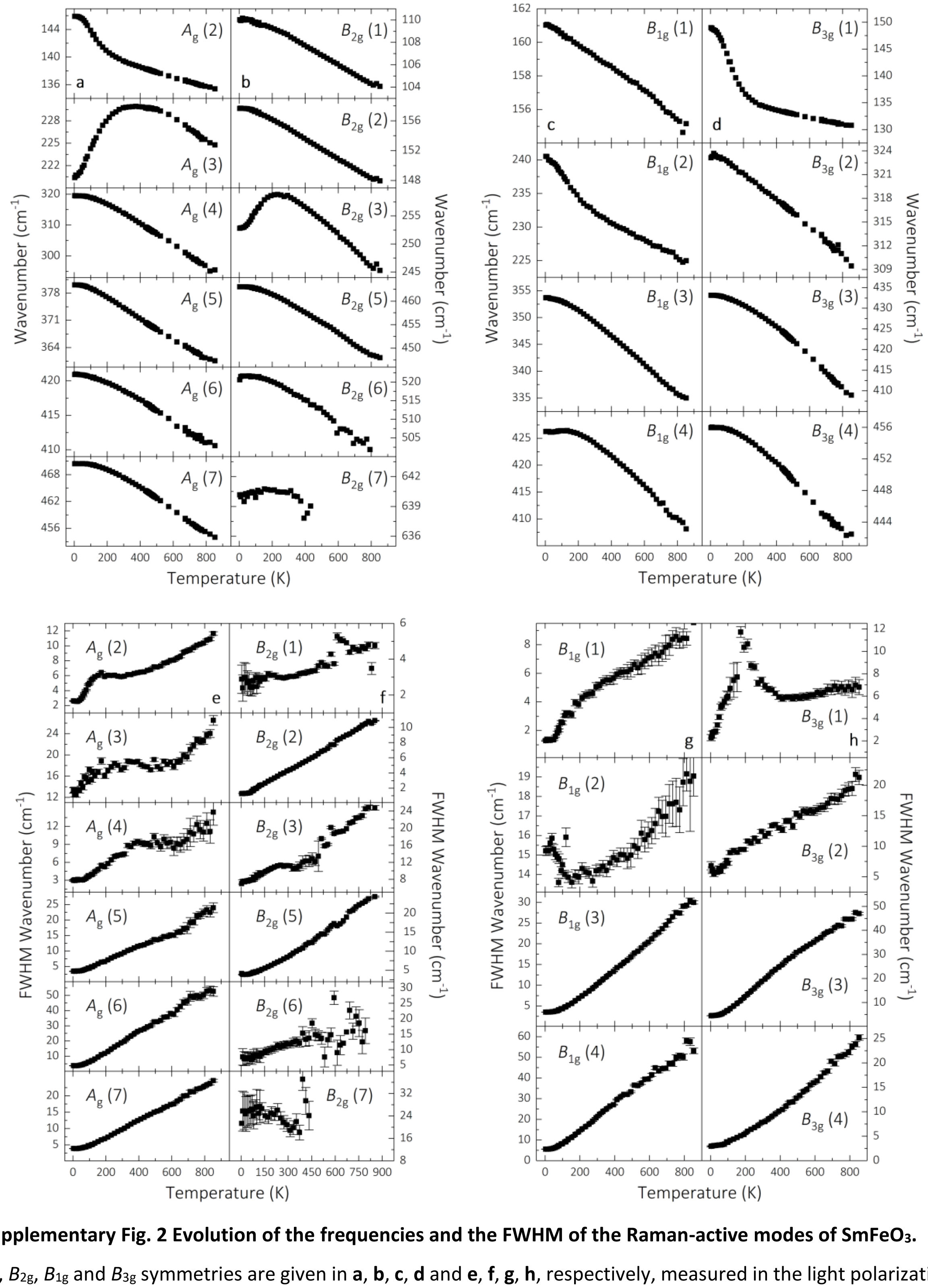

Supplementary Fig. 2 Evolution of the frequencies and the FWHM of the Raman-active modes of $SmFeO_3$. A_g, B_{2g}, B_{1g} and B_{3g} symmetries are given in **a**, **b**, **c**, **d** and **e**, **f**, **g**, **h**, respectively, measured in the light polarization settings as given in Supplementary Note 1. The modes are labeled following Ref. 1. The error bars refer to the fitting error. In a-d, the error bars are below the size of the data point. The quality of the spectra does not allow to trace the B_{2g}(7) mode reliably to high temperatures.

Supplementary Fig. 2 shows the evolution of the phonon frequencies and the full width half maxima (FWHM) with temperature. As discussed in the main text, we find strikingly anomalous behaviors in the frequency evolution with temperature of the $A_g(2)$, $A_g(3)$, $B_{2g}(3)$, $B_{1g}(2)$ and $B_{3g}(1)$ phonon modes. The FWHM, which are proportional to the inverse lifetime of the lattice vibrations, are even more sensitive to coupling effects. We find anomalies in the temperature evolutions of the FWHM for all low-frequency modes below 350 cm^{-1}. This means these modes are associated with Sm^{3+} motions. (The $B_{2g}(2)$ modes shows a peculiar behavior and needs a separate discussion.) With this information, we can anticipate that further phonons show an anomalous frequency evolution, though less strikingly, such as the $B_{1g}(1)$, $B_{2g}(1)$ and $B_{3g}(3)$ modes. The anomalous FWHM evolutions show comparable patterns. From high to low temperatures, the FWHM decline followed by a saturation-like regime in the range of T_N and T_{SR}. In cases, where strong anomalies are also visible in the frequency evolution, the saturation regime stretches down to about 200 K. In parts this is accompanied with an increase in FWHM. This is clearly seen for the $B_{1g}(1)$ phonon mode but can be anticipated for other modes too (see $A_g(2)$ or $B_{2g}(1)$). Subsequently, the FWHM show a steep decline (increase in case of the $B_{1g}(2)$ mode). A prominent the impact of the Sm^{3+}-Fe^{3+} spin interaction on phonons leads to a more pronounced plateau as the comparison with the phonon frequencies shows. Hence, the plateau can be interpreted as regime of phase competition, which is released as the FWHM re-decline.

High frequency modes (>350 cm^{-1}) do not show such anomalies. However, we find minor kinks at the magnetic ordering of the Fe^{3+} spins at T_N = 680 K (most clearly for $A_g(5)$, $A_g(6)$ but also in $A_g(7)$, $B_{1g}(3)$, $B_{3g}(3)$ modes). The absence of low temperature anomalies, yet the presents of the deviation at T_N underlines that both spin ordering phenomena are unrelated.

The $B_{2g}(2)$ mode is an interesting case. Despite a vibrational pattern motion of the Sm^{3+} ions, we do not observe any anomalous behavior. The $B_{2g}(2)$ mode primarily shows the displacement of Sm^{3+} ions in the *x*-direction. The other modes with anomalous evolutions include, however, other directional dependencies. This discrepancy between modes associated with different crystallographic directions, illustrates the strong anisotropic nature of the Sm^{3+}-Fe^{3+} coupling. This anisotropy is also found in X-ray diffraction measurements (see Supplementary Note 9).

Supplementary Note 3: New phonon bands in the low temperature regime

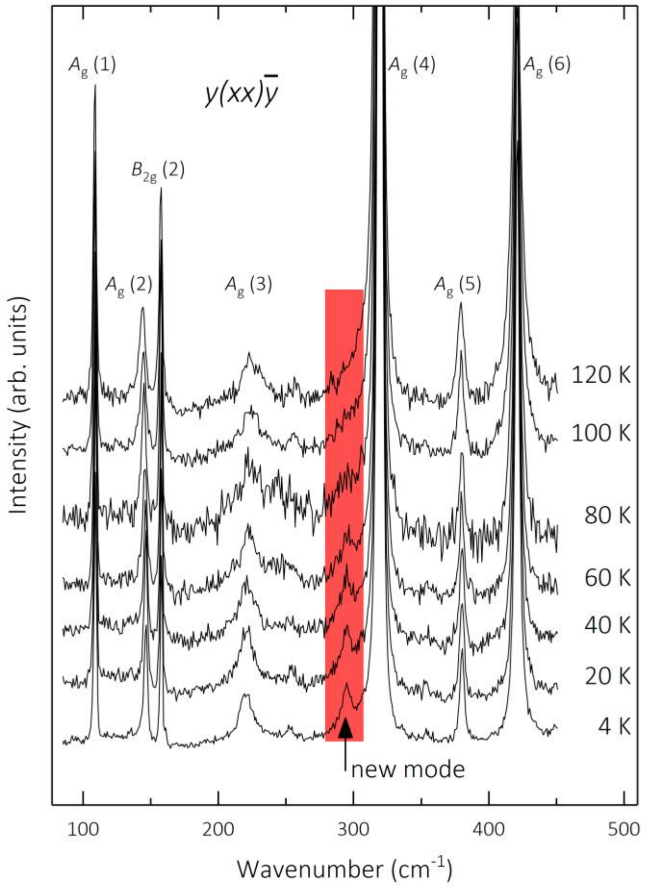

Supplementary Fig. 3 New phonon bands in the low temperature regime. Temperature dependent polarized Raman spectra of $SmFeO_3$ from 120 to 4 K measured in $y(xx)\bar{y}$ configuration. This configuration gives access to modes of A_g symmetry. Note, the B_{2g} mode of highest intensity is equally visible due to light polarization leakage caused by the use of a microscope objective and a potential minor misalignment of the sample. The red box indicates a new mode emerging at low temperatures. The Raman spectra were corrected for thermal occupation by (n + 1), where n is the Bose-Einstein occupation number. For better visibility, we normalized the spectra to the peak intensities of the mode at 380 cm^{-1}.

Figure 4 of the main text shows the emergence of new modes toward low temperatures for configurations, where the polarizations of incident and analyzed light are perpendicular to each other. However, new phonon modes are not limited to such crossed polarized settings of the incident and analyzed light. Supplementary Fig. 3 shows the emergence of a new phonon mode with decreasing temperature in the $y(xx)\bar{y}$-configuration, which gives access to phonons of A_g symmetry in the *Pnma* phase. We anticipate that more phonons modes emerge, yet below the resolution limit.

Supplementary Note 4: RUS analysis of the relaxation processes within the spin reorientation

Here, we analyze relaxation processes detected within the spin reorientation interval. The formal relationship between elastic moduli and acoustic loss for an anelastic process is given by $\tan\delta = \Delta\frac{\omega\tau}{(1+\omega^2\tau^2)}$, where $\tan\delta \approx Q^{-1}/\sqrt{3}$, ω is the angular measuring frequency and τ is the relaxation time for the loss mechanism[2,3]. In the case of a standard linear solid the parameter Δ is given by $\frac{(C_U - C_R)}{C_R}$ for (C_U - C_R)<< C_R, where C_U is the elastic modulus of the unrelaxed state and C_R is the elastic modulus of the relaxed state. The measuring frequency of ~1165 kHz for the data in Supplementary Fig. 4c was approximately constant across the reorientation transition interval so that deviations of Q^{-1} and f^2 from their values outside this interval, ΔQ^{-1} and Δf^2, should be linearly dependent if the other parameters remain constant. Supplementary Fig. 4d shows that a linear relationship does not hold, implying either that the relaxation time or the nature/density of defects, which move under external stress vary across the transition interval. There appear to be no other data in the literature for the elastic properties of $SmFeO_3$, but elastic softening across the same transition in $ErFeO_3$ and $YbFeO_3$ has been measured at frequencies of 30-170 MHz by pulse-echo ultrasonics and is accompanied by the same closely correlated increases in attenuation/loss. The pattern of softening is quite different, however[4,5]. Instead of a single rounded minimum in f^2 and maximum in Q^{-1}, there is a steep minimum in moduli and maximum in attenuation at both T_{SR1} and T_{SR2} transition temperatures. If the softening is again anelastic in origin, the differences point to changes in relaxation time.

The actual loss mechanism has not been determined but it must be significant that the anelastic anomalies occur only in the stability field of the intermediate state during the spin reorientation. Under the assumption of a continuous spin rotation between the space groups *Pn'ma'* and *Pn'm'a* (Γ_4 and Γ_2, respectively)[6,7] and on the basis of symmetry arguments alone, the intermediate state has to be monoclinic with space group $P2_1'/c'$ (Γ_{24}) based on the group theory program ISOTROPY[8] (and see Ref. 9). Our Raman spectroscopy data do not show any evidence for the existence of a long range ordered monoclinic state and X-ray diffraction measurements rule out the presence of measurable monoclinic distortions in $TmFeO_3$ and $ErFeO_3$[9,10]. However, splitting of NMR lines from Fe^{57} nuclei in both $ErFeO_3$ and $TmFeO_3$ has been interpreted as providing evidence for some structural distortion within the reorientation region[11,12]. The NMR sampling length scale would be purely local and, if present, domains with small local shear strains could give rise to the observed acoustic losses if their relaxation time in response to stress within an acoustic resonance varied in a symmetrical manner between T_{SR1} and T_{SR2}. Whether or not the locally distorted regions are monoclinic, increasing values of τ from 480 and 460 K to a maximum value at ~470 K, τ_{470}, would tend to give a single loss minimum and softening maximum at 470 K when measured at frequencies tending towards $\omega\tau_{470}$ = 1. Shorter relaxation times at 460 and

480 K, $\tau_{460,480}$, would give rise to minima in the elastic moduli and loss maxima at both 460 and 480 K when the measuring frequency tends to $\omega\tau_{460,480}$ = 1. In other words, a pattern of changing from a single broad peak to two sharper peaks would be observed with increasing frequency. Exactly this effect has been seen in analogous measurements of ac magnetic properties of $ErFeO_3$. The data of Shen *et al.*[13] for 5 kHz show two peaks in ac magnetic susceptibility, with correlated peaks in the loss, and a single broad peak in measurements made at 100 Hz.

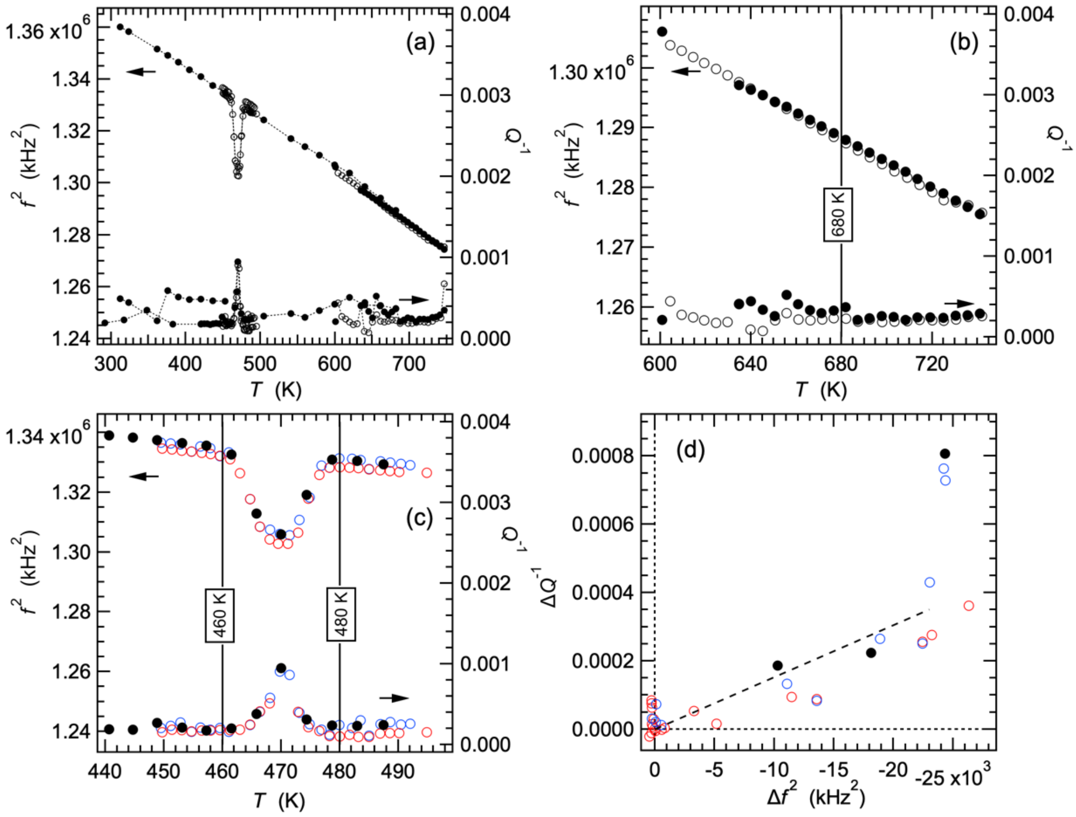

Supplementary Fig. 4 RUS analysis of the magnetic transitions. a Evolution of the square of the frequency, f^2, and inverse mechanical quality factor, Q^{-1}, for a single acoustic resonance peak in RUS spectra collected in heating (filled circles) and two cooling (red and blue open circles) sequences between room temperature and ~750 K. This resonance has a frequency near 1165 kHz at room temperature. **b** Expansion of the interval which includes T_N ~680 K, showing that there are no overt anomalies in f^2 or Q^{-1} accompanying the antiferromagnetic ordering transition. **c** Expansion of the temperature interval which includes the spin reorientation transition, showing correlation of elastic softening (decreases in f^2) and increases in acoustic loss (increasing Q^{-1}) between ~460 and ~480 K. **d** ΔQ^{-1} shows differences between measured Q^{-1} values in (c) and a linear baseline in the temperature interval ~460-480 K; Δf^2 represents differences between measured f^2 values and a linear baseline in the same interval. The broken line is a guide to the eye, excluding values for the midpoint of the temperature interval, ~470 K.

Supplementary Note 5: Primary RUS spectra tracing the spin reorientation transition

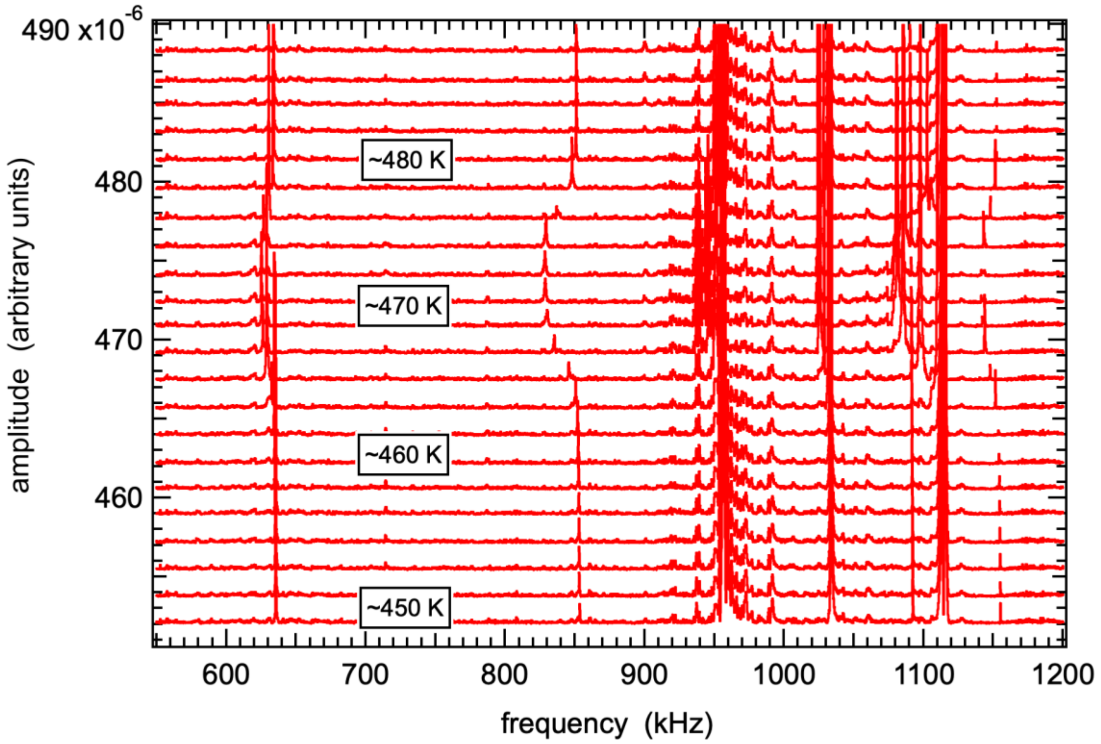

Supplementary Fig. 5 Example of primary RUS spectra of $SmFeO_3$ in a cooling sequence. Individual spectra have been offset up the *y*-axis in proportion to the temperature at which they were collected. Resonance peaks with frequencies that do not show any dependence on temperature, such as at ~990 and ~1060 kHz, are from the buffer rods. Resonance peaks from the sample show a shift to lower frequencies (elastic softening) followed by a return to higher frequencies (stiffening) between ~460 and ~480 K.

Supplementary Note 6: Relaxation during the spin reorientation of further elastic resonances

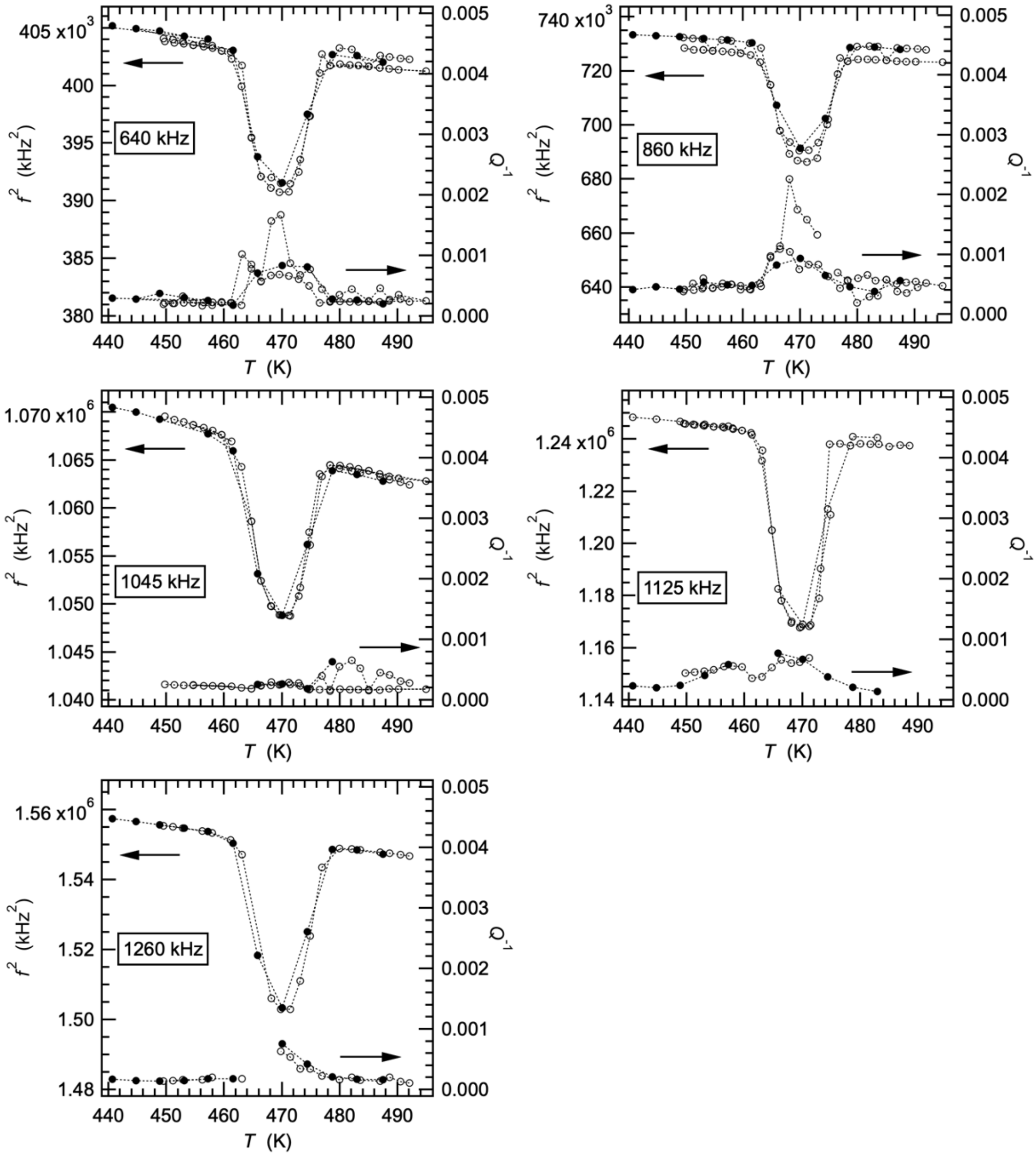

Supplementary Fig. 6 Relaxation during the spin reorientation of elastic resonances. Variations of f^2 and Q^{-1} from fitting of selected resonance peaks showing correlation of elastic softening (decreases in f^2) and increases in acoustic loss (increasing Q^{-1}) between ~460 and ~480 K. Filled circles = heating sequence, open circles = cooling sequences. The pattern of softening is the same for all the resonances. The data for Q^{-1} show much greater scatter, however, which is due primarily to interaction between resonances of the sample with resonances of the buffer rods, but the feature in common is an increase in the same temperature interval in each case.

Supplementary Note 7: Magnon evolution with temperature

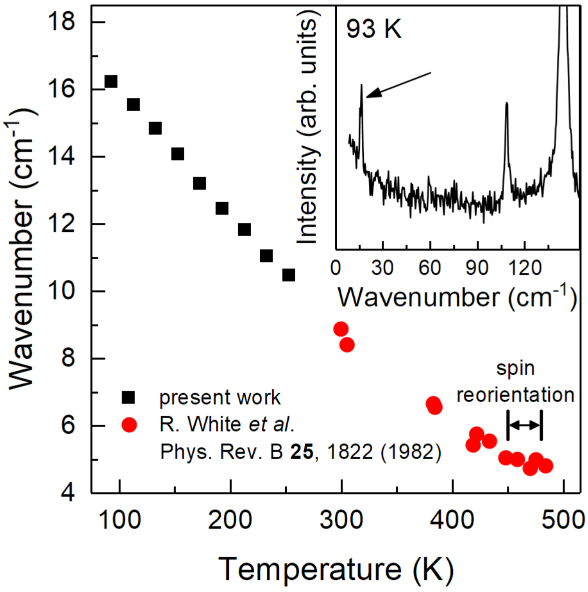

Supplementary Fig. 7 Evolution of the frequency of the A_g magnon mode with temperature. Values of the present work are given in black squares; red circles are taken from the work of White and co-workers[14]. Below room temperature, there is no evidence for any anomalous behavior. The insert shows the Raman spectrum at 93 K with the magnon mode indicated by the black arrow.

Supplementary Note 8: RUS analysis for the low temperature evolution

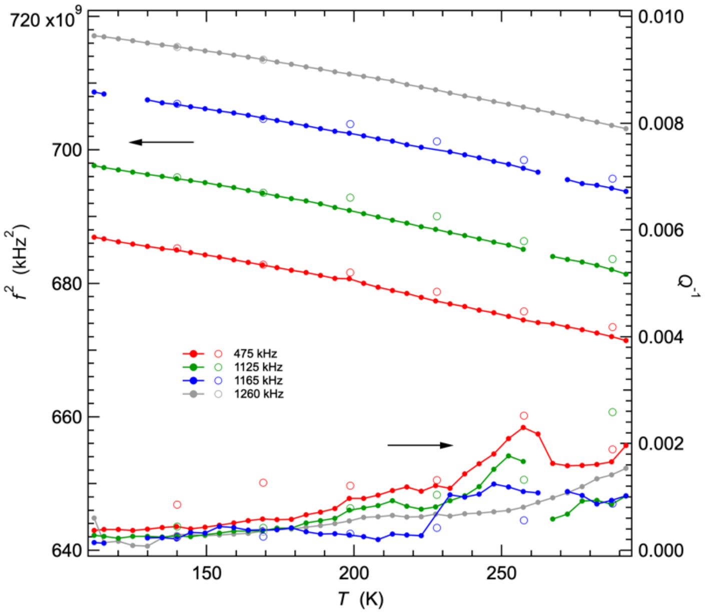

Supplementary Fig. 8 RUS analysis for the low temperature evolution of the elastic properties of $SmFeO_3$. Evolution of f^2 and Q^{-1} for selected acoustic resonances between room temperature and ~110 K. Open circles = cooling sequence, filled circles = subsequent heating sequence. Frequencies given in the legend correspond to the approximate frequency of each resonance at room temperature. Data for f^2 have been scaled along the *y*-axis by arbitrary factors for easy comparison between them. Smooth stiffening with falling temperature is shown by all four resonances, without obvious anomalies. Q^{-1} values increase with increasing temperature but show a hysteresis between decreasing and increasing temperature, and a weak peak between ~230 and ~275 K for the resonances with frequencies near 475, 1125 and 1165 kHz.

Variations of f^2 between ~300 K and ~110 K show the normal smooth increase with falling temperature, without any overt anomalies, as would be expected for elastic moduli[15], although there is a small difference between cooling and heating at about 190 K and above (Supplementary Fig. 8). Q^{-1} values show a trend of slight increases with increasing temperature, with a divergence above ~180 K and a tendency for values from resonances with the lowest frequency to increase to the greatest extent. There is also a broad, weak peak in Q^{-1} between ~230 and ~270 K for the resonances with frequencies near 475, 1125 and 1165 kHz. These subtle variations are at the limit of experimental sensitivity, however. Evidence for a magnetic glass transition with a zero frequency freezing temperature of ~120 K has recently emerged with, in particular, a strong frequency dependent loss peak in ac magnetic data collected in the frequency range 0.01-1 kHz[16,17]. If the glassy freezing of moments is accompanied by even a small change in shear strain, a frequency dependent peak in the acoustic loss would also be expected. Indeed, we observe anomalous lattice-parameter change (see Supplementary Note 9). Extrapolation of fits to the ac magnetic loss data at 0.01-1 kHz of the type shown in Fig. 4 of De *et al.* would give maxima in loss parameters at ~250 K for 100 kHz to ~330 K for 1 MHz[17]. Thus it is possible

that the weak peak in Q^{-1} near 250 K is indicative of a magnetoelastic component to the cluster glass transition at ~120 K. A further indicator of magnetoelastic coupling is the correlation of hysteresis in values of f^2 between about 190 K and room temperature. Hysteresis was reported in DC magnetization results for the same interval by De *et al*. and was interpreted in terms of a glassy magnetic state[17].

Supplementary Note 9: Temperature evolution of the lattice parameters

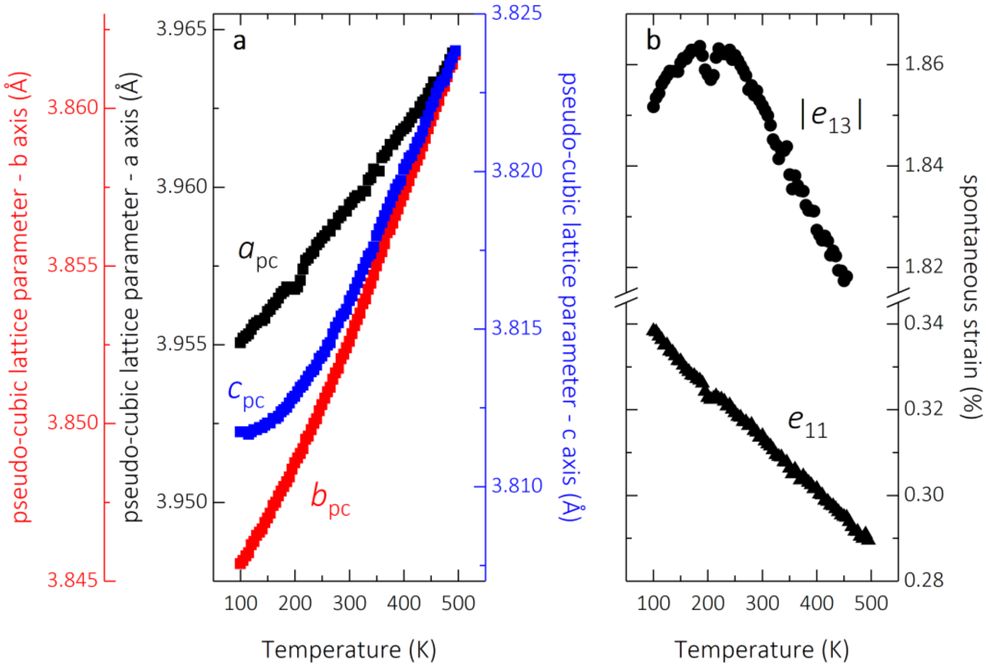

Supplementary Fig. 9 Temperature evolution of the lattice parameters and the spontaneous strain.
a Temperature evolution of the pseudo-cubic lattice parameters a_{pc}, b_{pc}, c_{pc} of $SmFeO_3$ extracted from single-crystal synchrotron X-ray diffraction measurements. **b** Temperature evolution of the independent components e_{11}, e_{13} of the spontaneous strain tensor as discussed in the text.

To understand further the impact of the Sm^{3+}-Fe^{3+} spin interaction on the crystal structure, we turn to X-ray diffraction measurements. The single crystal X-ray diffraction experiment was performed at the European Synchrotron Radiation Facility (ESRF) on the Swiss-Norwegian beamline, BM01. The beam was monochromatic with a wavelength of 0.99099 Å. The beamline is equipped with a Pilatus 2M area pixel-counting detector. The data was collected with attenuated primary beam during single 360° rotation scans around the φ-axis with a 1° step size per frame. The determination of the crystal orientation, indexing and integration of the Bragg peaks were carried out using the package XDS. The temperature was controlled using an Oxford Cryostream system. Supplementary Fig. 9a shows the evolution of the pseudo-cubic lattice parameters of $SmFeO_3$ with temperature. The lattice constants of the *a* and *b* axes show a homogeneous evolution with temperature. The *c*-axis, however, clearly shows an anomalous deviation toward low temperatures.

To reveal the anomaly more clearly, it is beneficial to calculate the components of the spontaneous strain tensor from the lattice parameters. As described e.g. in Refs. 18,19 for the *Pnma* structure, the tensor reduces to the form:

$$\begin{pmatrix} e_{11} & 0 & e_{13} \\ 0 & -2e_{11} & 0 \\ e_{13} & 0 & e_{11} \end{pmatrix},$$

with the two independent symmetry-breaking components e_{11} and e_{13}. The former scales with the elongation along the longer *b*-axis and the latter is a shear strain in the *ac*-plane. Both components are shown in Supplement Fig. 9b. The evolution of the e_{11} is nearly linear over the full temperature range, whereas the shear strain e_{13} shows a very clear anomaly with a maximum at 200 K that coincides with the reported phonon anomalies. The gradual nature of the deviation mirrors the continuous emergence of the Sm^{3+}-Fe^{3+} spin interaction. The fact that only the shear strain is affected underlines the anisotropic character of the Sm^{3+}-Fe^{3+} spin interaction. This also matches our observations in the anomalies of the phonon modes as discussed in Supplementary Note 2. Note, the small dip in the length of the *a*-axis near 220 K is most likely an artifact of the measurement.

Supplementary References

1. Weber, M. C. *et al.* Raman spectroscopy of rare-earth orthoferrites $RFeO_3$ (R = La, Sm, Eu, Gd, Tb, Dy). *Phys. Rev. B* **94**, 214103 (2016).
2. Nowick, A. S. & Berry, B. S. *Anelastic Relaxation in Crystalline Solids*. (Academic Press, 1972).
3. Carpenter, M. A., Buckley, A., Taylor, P. A. & Darling, T. W. Elastic relaxations associated with the $Pm\bar{3}m$ – $R\bar{3}c$ transition in $LaAlO_3$: III. Superattenuation of acoustic resonances. *J. Phys. Condens. Matter* **22**, 035405 (2010).
4. Gorodetsky, G. & Lüthi, B. Sound-Wave—Soft-Mode Interaction near Displacive Phase Transitions: Spin Reorientation in $ErFeO_3$. *Phys. Rev. B* **2**, 3688–3698 (1970).
5. Dan'shin, N. K. *et al.* Dynamic properties of $YbFeO_3$ in the vicinity of an orientational phase transition. *Sov. Phys. JETP* **66**, 1227–1232 (1987).
6. White, R. L. Review of recent work on the magnetic and spectroscopic properties of the rare-earth orthoferrites. *J. Appl. Phys.* **40**, 1061–1069 (1969).
7. Deng, G. *et al.* The magnetic structures and transitions of a potential multiferroic orthoferrite $ErFeO_3$. *J. Appl. Phys.* **117**, 164105 (2015).
8. Stokes, H. T., Hatch, D. M. & Campbell, B. J. ISOTROPY Software Suite. (2007).
9. Tsymbal, L. T. *et al.* Magnetic and structural properties of spin-reorientation transitions in orthoferrites. *J. Appl. Phys.* **101**, 123919 (2007).
10. Tsymbal, L. T., Kamenev, V. I., Bazaliy, Y. B., Khara, D. A. & Wigen, P. E. Structural properties of $ErFeO_3$ in the spin-reorientation region. *Phys. Rev. B* **72**, 052413 (2005).
11. Doroshev, V. D. *et al.* NMR investigation of spin reorientation nature in thulium orthoferrite. *Phys. Stat. Sol.* **51**, K31 (1972).
12. Kovtun, N. M., Karnachev, A., Solov'ev, E. E., Chervonenkis, A. Y. & Shemyakov, A. A. NMR study of spin reorientation in the weak ferromagnet $ErFeO_3$. *Sov. Phys. Solid State* **14**, 1856 (1973).
13. Shen, H. *et al.* Magnetic field induced discontinuous spin reorientation in $ErFeO_3$ single crystal. *Appl. Phys. Lett.* **103**, 192404 (2013).
14. White, R. M., Nemanich, R. J. & Herring, C. Light scattering from magnetic excitations in orthoferrites. *Phys. Rev. B* **25**, 1822–1836 (1982).
15. Varshni, Y. P. Temperature Dependence of the Elastic Constants. *Phys. Rev. B* **2**, 3952–3958 (1970).
16. Warshi, M. K. *et al.* Cluster Glass Behavior in Orthorhombic $SmFeO_3$ Perovskite: Interplay between Spin Ordering and Lattice Dynamics. *Chem. Mater.* **32**, 1250–1260 (2020).
17. De, C., Nayak, A. K., Nicklas, M. & Sundaresan, A. Magnetic compensation-induced sign reversal of exchange bias in a multi-glass perovskite $SmFeO_3$. *Appl. Phys. Lett.* **111**, 182403 (2017).
18. Carpenter, M. A., Becerro, A. I. & Seifert, F. Strain analysis of phase transitions in (Ca,Sr)TiO_3 perovskites. *Am. Mineral.* **86**, 348–363 (2001).
19. Yokota, H. *et al.* Direct evidence of polar nature of ferroelastic twin boundaries in $CaTiO_3$ obtained by second harmonic generation microscope. *Phys. Rev. B* **89**, 144109 (2014).
